# Drivers influencing antibiotics use in pediatric wards across five public hospitals in Zanzibar: a WHO multiple point prevalence survey

**DOI:** 10.3389/frabi.2026.1874316

**Published:** 2026-07-30

**Authors:** Faiza Mohamed Juma, Abrahaman Said Msellem, Stanley Mwita, Muhiddin Omar, Khadija Omar, Daniel Joshua, Ahlam Amour, Hafidh Hassan, Salma Hashoul, Muhiddin A. Mahmoud, Simon Kühnert, Mayassa Salum Ally, Ali Said, Jyoti Joshi, Fabian Maza, Brecht Ingelbeen, Eveline Thobias Konje, Anja Poulsen, Stine Lund, Jeremiah Seni

**Affiliations:** 1 Department of Epidemiology and Biostatistics, Catholic University of Health and Allied Sciences, Mwanza, Tanzania; 2 Pediatric Department, Mnazi Mmoja Hospital, Zanzibar, Tanzania; 3 Ministry of Health, Zanzibar, Tanzania; 4 Department of Pharmaceutics and Pharmacy Practice - School of Pharmacy. Catholic University of Health and Allied Sciences, Mwanza, Tanzania; 5 Zanzibar Health Research Institute (ZAHRI), Zanzibar, Tanzania; 6 Zanzibar Agriculture and Livestock Research Institute (ZALIRI), Zanzibar, Tanzania; 7 Health Improvement Project Zanzibar (HIPZ), Zanzibar, Tanzania; 8 Public Health Laboratory Ivo de Carneri (PHL-IdC), Pemba, Tanzania; 9 International Center for Antimicrobial Resistance Solution (ICARS), Copenhagen, Denmark; 10 Institute of Tropical Medicine, Antwerp, Belgium; 11 Global Health Unit, Department of Paediatrics and Adolescent Medicine, Copenhagen University Hospital Rigshospitalet, Copenhagen, Denmark; 12 Department of Paediatrics, Copenhagen University Hospital North Zealand, Hillerød, Denmark; 13 Department of Microbiology and Immunology - Weill-Bugando School of Medicine, Catholic University of Health and Allied Sciences, Mwanza, Tanzania

**Keywords:** antibiotics, antimicrobial stewardship, pediatric, WHO-PPS, Zanzibar

## Abstract

**Background:**

Strengthening antimicrobial stewardship (AMS) programs is a major public health strategy to reduce inappropriate antibiotic use (AMU) and averting antibiotic resistance (AMR). Evidence on AMU and AMS program implementation is widely documented in Tanzania mainland, consistent with previous studies done and other low-and middle-income countries. However, there is limited information on AMU and AMS programs to guide the implementation of the Zanzibar Action Plan on AMR. The objective of this study was to determine the prevalence of AMU, identify factors associated with AMU, and to assess the performance of AMS programs in pediatric wards across hospitals in Zanzibar.

**Methods:**

This repeated cross-sectional study employed the World Health Organization Point Prevalence Survey (WHO-PPS) tool at two time points (February to April 2024 and December 2024 to May 2025), complemented by a one-time WHO-Health Care Facility Core Element Indicators Tool conducted in April 2024 to assess AMS performance. The WHO-PPS study population was children ≤13 years admitted to pediatric wards in five public hospitals in Zanzibar. Healthcare providers and AMS team members reported on Core Element Indicators. The WHO-PPS data were analyzed using descriptive statistics and multivariate modified Poisson regression, while AMS performance was computed as percentage scores per hospital.

**Results:**

Of 943 paediatric patients, 826 (87.6%) received at least one antibiotic. Gentamicin, ampicillin, and ceftriaxone were the most commonly prescribed antibiotics. Watch-category antibiotics accounted for the majority of prescriptions (53.8%), followed by Access (45.8%) and Reserve (0.4%). Community-acquired infections were the predominant indication for antibiotic use, 60.4%, with pneumonia and sepsis being the most common diagnoses. Bacteriological culture testing was performed in only 10.9% of patients, while adherence to national treatment guidelines was 77.8%. Significant predictors of AMU included hospital tier (regional: aPR 1.39, 95%CI 1.30-1.50; and district level: aPR 1.35, 95%CI 1.26-1.46); and higher disease severity shown by ultimately fatal McCabe score, aPR 1.20, 95%CI: 1.07-1.33). The AMS program performance was below the 50% functionality threshold, with low scores observed in leadership, accountability, monitoring and surveillance, and reporting and feedback, alongside limited progress in the establishment of DTC/IPC/AMC committees and implementation of AMS actions.

**Conclusions:**

Antibiotic use among admitted children was markedly prevalent, notably by the Watch category of antibiotics, and limited reliance on bacteriological culture and suboptimal AMS program performance across hospitals in Zanzibar. These findings highlight important opportunities to strengthen AMS programs and diagnostic practices. Future research should evaluate the appropriateness of antibiotic prescribing and explore barriers to effective AMS implementation across hospitals in Zanzibar.

## 1 Introduction

Antibiotics remain central to treating infectious diseases, but their misuse has driven the global rise of antimicrobial resistance (AMR), especially among vulnerable pediatric populations in low- and middle-income countries (LMICs) (Romandini et al., 2021; Kassa and Al-Sayidi, 2023; Salam et al., 2023). Strengthening antimicrobial stewardship (AMS) programs is a key strategy to reduce inappropriate antibiotic use and AMR (Davey et al., 2017). The World Health Organization (WHO) has emphasized the essential role of AMS in ensuring appropriate antibiotic use, improving patient outcomes, and combating AMR (World Health Organization, 2019). While AMS broadly addresses all antimicrobial agents, including antivirals, antifungals, and antiparasitics, this study focused specifically on antibiotic use (AMU). Admitted patients are more likely to be prescribed antibiotics, and the prevalence of AMU has been shown to vary within hospitals, hospital tiers and the patient populations from 40.9% (UK), 44.3% (Canada), 62.0% (Indonesia), 62.3%(Tanzania), and (64.0%), in a systematic review and meta-analysis involving over 26, 000 patients from 10 countries in sub-Saharan Africa countries (Gharbi et al., 2016; Seni et al., 2020; Blackburn et al., 2021; Limato et al., 2021; Boltena et al., 2024).

Appropriate antibiotic usage is a major challenge in pediatric settings due to the vulnerability of children, the complexity of their medical conditions, and the need for accurate dosing and treatment protocols (Branstetter et al., 2021). Antibiotic prescribing is a complex process influenced by multiple factors, including clinical guidance, time pressure, pharmaceutical companies’ influence, financial considerations, prior experience among professionals, prescribers’ behaviors, and patient expectations (Kasse et al., 2024). Consequently, inappropriate use often arises from the combined effects of prescriber, patient, and healthcare facility-related factors, underscoring the need for a comprehensive approach that addresses all these dimensions (Liu et al., 2019). Zanzibar, like any other LMICs, faces significant challenges in establishing effective AMS programs due to resource constraints and the multisectoral nature of the AMR burden (World Health Organization, 2015a; Revolutionary Government of Zanzibar, 2019; World Health Organization and World Health Organization. Regional Office for South-East Asia, 2022). Investments in infrastructure, training, and policy frameworks are therefore crucial for the successful implementation of AMS programs (Gebretekle et al., 2018). Assessing the performance of AMS programs is essential for guiding effective interventions, identifying gaps, and supporting ongoing quality improvement. In 2019, Zanzibar adopted the Zanzibar Action Plan (ZAP) on AMR, which aligns with the WHO Global Action Plan (World Health Organization, 2015b; Revolutionary Government of Zanzibar, 2019), but local evidence on pediatric AMU and AMS performance remains scarce.

Despite the implementation of the Zanzibar Action Plan on AMR (Revolutionary Government of Zanzibar, 2019), there is still limited evidence on antibiotic use practices, factors influencing antibiotic prescribing, and the implementation of AMS programmes among hospitalized paediatric patients in Zanzibar. This lack of local evidence limits healthcare facilities’ and policymakers’ ability to identify key drivers of antibiotic use, monitor stewardship performance, and design targeted interventions to improve antibiotic prescribing practices. Given the high burden of antibiotic use among paediatric inpatients and the growing threat of AMR (Gerber et al., 2021; Romandini et al., 2021; Salam et al., 2023), generating context-specific evidence is essential to support the implementation and evaluation of AMS strategies across Zanzibar’s healthcare system.

The WHO Point Prevalence Survey (WHO-PPS) is a validated tool for monitoring hospital AMU to inform AMS interventions (Chikowe et al; Gharbi et al., 2016; Pauwels et al., 2021) and is used to monitor the strategic objective on the rational use of antibiotics in the countries’ respective National Action Plans, and to report AMU trends to the WHO-Global Antimicrobial Resistance and Use Surveillance System (GLASS). The previous WHO-PPS studies conducted in Tanzania mainland showed high empirical antibiotic usage (44% to 62.3%), and the most prescribed antibiotics were ceftriaxone, metronidazole, and ampicillin. The main factors associated with antibiotic use were age <2 years, admission to surgical wards, and admission to pediatric wards (Horumpende et al., 2020; Seni et al., 2020; Katyali et al., 2023). No comparable evidence exists for Zanzibar, and also the Zanzibar health system and the Ministry of Health are independent from mainland Tanzania. To complement the WHO-PPS tool, which addresses AMU at the patient level, the WHO developed another tool to evaluate the AMS programs at the health care facility level, which is called the WHO-Health Care Facility (HCF) Core Element Indicators Tool (World Health Organization, 2018; World Health Organization, 2019). This tool has eight AMS core elements, which can be monitored over time to assess facilities’ performance and guide corrective actions. For example, pre- and post-AMS intervention surveys across 32 hospitals in five countries in Africa showed increased scores for AMS core elements from 34% to 79% at the baseline to 58% to 92% at the endline between 2022 and 2023 (Wesangula et al., 2025).

Our study was conducted to identify AMS implementation gaps in priority paediatric patients admitted across three tiers of health care facilities (i.e., district, regional, tertiary levels), guiding implementation of the ZAP. Triangulating AMU practices, factors influencing AMU and AMS interventions, measured using the two WHO tools, could yield evidence-based contextualised recommendations to inform AMS policy.

## 2 Materials and methods

### 2.1 Study design, duration, and setting

Repeated cross-sectional analytical studies were conducted using the WHO-PPS tool at two time points (February–April 2024 and December 2024–May 2025), and complemented by a one-time WHO-AMS assessment in April 2024. This study was nested within the Zanzibar ANtibiotic Treatment Of Childhood Infections To Improve Health Outcomes Project (ZAN-TOTO, https://icars-global.org/projects/zanzibar_zantoto/). This study assessed AMU and AMS at baseline across five public hospitals in Zanzibar (three at the district level, one regional level, and one tertiary level). In Unguja Island, the hospitals involved were Mnazi Mmoja Hospital (MMH, tertiary-level) and Kivunge Hospital (KVH, district); in Pemba Island, Abdulla Mzee Hospital (AMH, regional), Vitongoji District Hospital (VDH, district), and Kinyasini District Hospital (KDH, district).

### 2.2 Study population

The study population included all paediatric inpatients (≤13 years of age) admitted to selected public hospitals in Zanzibar during the WHO-PPS data collection periods. All patients present in the paediatric wards on the survey days were eligible for inclusion and were assessed according to the WHO-PPS methodology. Admitted children with incomplete data in their medical files, children admitted for day-care surgeries (like circumcision) or daycare services (like dialysis or chemotherapies) were excluded. The WHO-HCF AMS assessment included health care providers in pediatric wards and AMS team members (i.e. chairperson and secretary of the team/committee) at the five selected hospitals.

### 2.3 Sample size and strategy

The sample size was determined using the WHO-PPS methodology, which recommends including all eligible inpatients in hospitals with <500 beds, admitted in selected wards before 8:00 AM on the day of the survey. Since all five hospitals had <500 beds during the survey periods, all eligible children admitted to pediatric wards were included, yielding a total of 973 participants. After excluding 30 records with missing data, a total of 943 eligible participants were included in the final analysis. For the WHO-HCF AMS assessment, purposive sampling was applied to select two key stewardship team members (chairperson and secretary) in each hospital (World Health Organization, 2018; World Health Organization, 2019).

#### 2.3.1 Data collection

Trained data collectors collected WHO-PPS data from medical records at two time points, including hospital, ward, patient, indication, and antibiotic information. Only antibiotics administered through oral, parenteral, rectal, or inhalation routes were included. AMS core element indicators assessment data were collected through interviews and through document review (World Health Organization, 2018; World Health Organization, 2019). Data were entered into standardized MS Excel templates. Study Variables captured from each tool were:

#### 2.3.2 From the WHO-PPS tool

hospital, ward, patient (age, gender, prior admission, surgery since admission, and transfer status), prescriber rank, antibiotic indication, and prescribed antibiotic data. These were used to assess prevalence, factors associated with AMU, and Access Watch and Reserve (AWaRe) categories of antibiotics’ conformity to the WHO-targets and Zanzibar Standard Treatment Guidelines.

From the WHO-HCF AMS tool: presence of Drug Therapeutic Committee (DTC), Infection Control Committee (IPC) or AMS team; DTC functionality; Leadership commitment; Accountability and responsibility; AMS actions; Education and training; Monitoring and surveillance, and Reporting feedback within healthcare facility. These were used to evaluate AMS performance.

#### 2.3.3 Study outcomes

The primary outcomes were the prevalence of AMU and the percentage score performance of AMS programs in five public hospitals. Secondary outcomes included factors influencing AMU, conformity to WHO-AWaRe categories, and other AMS key actions.

### 2.4 Data analysis

Data were analyzed using STATA version 17.0. Descriptive statistics were used to determine the prevalence of AMU, expressed as frequencies and percentages. Association between categorical variables and AMU was first assessed with the Pearson chi² test, followed by bivariate modified Poisson regression to estimate the prevalence ratio (PR), with 95% confidence intervals (CIs), with p-values <0.2 used for variable selection. Multivariate modified Poisson regression was then applied to identify independent predictors of AMU, with aPR and 95% CIs reported, and p-values < 0.05 considered statistically significant. Although the modified Poisson regression in STATA reports incidence rate ratios (IRRs), given the cross-sectional design of this study, these estimates were interpreted as prevalence ratios (PR). Hospital identity was initially modelled as a fixed effect to control for differences across facilities, but was later excluded due to minimal variation and risk of bias.

The WHO-AMS assessment tool was used to extract AMS performance information, which includes predefined indicators and scoring criteria for each core element, and included eight core elements (World Health Organization, 2019). To evaluate the performance of the AMS program across the five hospitals, we described a score for each of each WHO-AMS performance core element and a total percentage score for each hospital. The 8 core elements are: the presence of a DTC, IPC, or AMS team; DTC functionality; Leadership commitment; Accountability and responsibility; AMS actions; Education and training; Monitoring and surveillance; and Reporting feedback within the healthcare facility. Each core element has subcomponents, whereby each has a grade score from 0 to 4. The percentage for each core element is calculated by the summation of the total grade score of each core element divided by the total score for all subcomponents times by a hundred. The score for each core element was 0%-100%, and the final score is the cumulative summation of the scores of each core element. The final AMS performance interpretations were:

- The AMS score between 0.0% and 49.9% means the AMS program was either non-functional or operating poorly, and that it needed to be implemented or strengthened.
- The AMS score of 50%–79.9% means that the AMS program was only partially operational and required support to be strengthened.
- The AMS score between 80% and 100% means that the AMS program was fully established and operating as intended, but it still needed ongoing assistance to be sustainable.

## 3 Results

### 3.1 Baseline demographic and clinical information of patients enrolled

A total of 943 pediatric patients were enrolled across five hospitals, of which 333(35.35) were admitted to tertiary-level care. Infants (<12 months) accounted for nearly two-thirds of admissions 633(67.2%). 854 (90.6%) patients had a peripheral vascular catheter in place (**Table 1**).

**Table 1:** Socio-demographic and clinical information of enrolled participants in five public hospitals in Zanzibar (N = 943).

Variable	Category	Frequency (n)	Percentage (%)
Hospital	Mnazi Mmoja Tertiary Hospital	333	35.3
	Vitongoji District Hospital	244	25.9
	Abdulla Mzee Regional Hospital	163	17.3
	Kivunge District Hospital	138	14.6
	Kinyasini District Hospital	65	6.9
Gender	Male	537	56.9
	Female	406	43.1
Age group (months)	>60	71	7.5
	13-60	239	25.3
	2-12	307	32.6
	≤1	326	34.6
Surgery since admission	Yes	39	4.1
	No	904	95.9
Peripheral vascular catheter	Yes	854	90.6
	No	89	9.4
Transfer from other hospitals	Yes	139	14.7
	No	804	85.3
Admission in the past 90 days	Yes	19	2.0
	No	924	98.0

### 3.2 Prevalence of antibiotic use among pediatric patients admitted to five public hospitals in Zanzibar

The prevalence of antibiotic use among pediatric inpatients was 87.6% (95% CI: 85.3%-89.6%) across all five hospitals, with markedly higher AMU in district hospitals, ranging (92.0%-96.9%). Prevalence of AMU was 99.4% (95% CI: 97%-99%) at the regional hospital and 71.8% (95% CI: 67%-77%) at the tertiary facility (**Table 2**).

**Table 2:** Prevalence of antibiotic use among admitted pediatric patients across five public hospitals in Zanzibar.

Hospital	No of patients	No of patients on antibiotics	Prevalence of antibiotic use (%)	95% CI (%)
Mnazi Mmoja Hospital	333	239	71.8	67-77
Vitongoji District Hospital	244	235	96.3	93-98
Abdulla Mzee Hospital	163	162	99.4	97-99
Kivunge District Hospital	138	127	92.0	86-96
Kinyasini District Hospital	65	63	96.9	89-99
All	943	826	87.6	85-90

### 3.3 Indications for antibiotic use and diagnoses among pediatric inpatients across five public hospitals in Zanzibar

A total of 882 indications for antibiotic use were recorded. Community-acquired infections (CAI) were the most common (60.4%), followed by Hospital-acquired infections (HAI) (20.6%), while prophylactic use was less frequent (10.2%). The highest proportion of CAI-related antibiotic use was observed at Vitongoji District Hospital (VDH), with 186 (78.5%), followed by Abdulla Mzee Regional Hospital (AMH), with 122 (75.3%). HAI was the second most common indication, accounting for 182 prescriptions (20.82%) (**Table 3**). Across the five hospitals, the main diagnoses for antibiotic use were pneumonia (27.3%) and clinical sepsis (18.0%), while (33.7%) of prescriptions were given for non-treatment purposes (**Supplementary File 1**).

**Table 3:** Indications for antibiotic use among pediatric inpatients across five public hospitals in Zanzibar.

Indication for antibiotics	MMH n (%)	VDH n (%)	AMH n (%)	KVH n (%)	KDH n (%)	Total N (%)
Community-Acquired Infection	118 (41.6)	186 (78.5)	122 (75.3)	72 (53.7)	35 (53.9)	533 (60.4)
Hospital-acquired infections	58 (20.4)	33 (13.9)	28 (17.3)	43 (32.1)	20 (30.8)	182 (20.6)
Medical Prophylaxis	71 (25.0)	3 (1.3)	8 (4.9)	7 (5.2)	1 (1.5)	90 (10.2)
Surgical Prophylaxis	11 (3.9)	4 (1.7)	2 (1.3)	1 (0.8)	0 (0.0)	18 (2.0)
Other	26 (9.2)	11 (4.6)	2 (1.2)	11 (8.2)	9 (13.9)	59 (6.7)
Total	284 (100.0)	237 (100.0)	162 (100.0)	134 (100.0)	65 (100.2)	882 (100.0)

MMH, Mnazi Mmoja Tertiary Hospital; KVH, Kivunge District Hospital; VDH, Vitongoji District Hospital; AMH, Abdulla Mzee Regional Hospital; KDH: Kinyasini District Hospital.

### 3.4 Distribution of antibiotic prescriptions and the WHO AWaRe categories

A total of 1, 385 antibiotic prescriptions were recorded. The most frequently used antibiotics were gentamicin 494 (35.7%), ampicillin 440 (31.8%), and ceftriaxone 223 (16.1%), altogether 1157 (83.6%) of prescriptions (**Supplementary File 2**). Watch-group antibiotics were more commonly used 745 (53.8%) than Access-group 635 (45.8%), and Reserve-group antibiotics 5 (0.4%). The proportion of the Access category of antibiotics was below the WHO target of >60.0%, while the Watch category of antibiotics substantially exceeded the ≤28% target. Reserve category of antibiotics use was minimal and conforming to the WHO-target of <12.0% (**Table 4**). Antibiotic prescriptions were distributed across AWaRe categories according to national target levels defined for different levels of care.

**Table 4:** WHO AWaRe classification of antibiotics in pediatric departments across five public hospitals in Zanzibar.

AWaRe Classification	MMH n (%)	VDH n (%)	AMH n (%)	KVH n (%)	KDH n (%)	TOTAL N (%)	WHO Target (%)
Access	216(50.4)	181(46.5)	94(39.7)	105(45.3)	39(39.8)	635(45.8)	>60.0
Watch	208(48.4)	208(53.5)	143(60.3)	127(54.7)	59(60.2)	745(53.8)	≤28.0
Reserve	5 (1.2)	0(0.0)	0(0.0)	0(0.00)	0(0.00)	5(0.4)	≤12.0
**Total**	429(100.0)	389(100.0)	237(100.0)	232(100.0)	98(100.0)	1385(100.0)	100.0%

MMH, Mnazi Mmoja Tertiary Hospital; KVH, Kivunge District Hospital; VDH, Vitongoji District Hospital; AMH, Abdulla Mzee Regional Hospital; KDH, Kinyasini District Hospital. The colors correspond to the WHO AWaRe classification of antibiotics: Green = Access (first- and second-choice antibiotics with lower resistance potential), Yellow/Amber = Watch (antibiotics with higher resistance potential that should be prioritized for stewardship and monitored), and Red = Reserve (last-resort antibiotics reserved for treatment of confirmed or suspected multidrug-resistant infections).

### 3.5 Assessment of other key AMS indicators

Culture and antimicrobial susceptibility testing (AST) were rarely performed, with only 89 (10.9%) of prescriptions supported by culture results. Among the tests, blood cultures were 80 (95.2%), and urine was 1 (1.2%). Uptake of AST was highest at VDH 72 (30.4%), while it was minimal at MMH 13 (4.6%), KVH 3 (2.2%), and AMH 1(0.6%) hospitals, and absent at KDH 0 (0.0%). Adherence to the Zanzibar Standard Treatment Guidelines was 77.8%, while 18.1% were non-adherent to STG and smaller proportions were un-assessable (3.1%) or undocumented (1.0%). Most prescriptions were issued by general practitioners 1074 (77.7%), with only 208 (15.0%) from specialists and a smaller share from other cadres 101 (7.3%). The parenteral route predominated (98.6%), while oral administration was low (1.4%).

### 3.6 Factors influencing antibiotic use among pediatric inpatients in five public hospitals in Zanzibar

Significant predictors of antibiotic use were hospital tier and disease severity. The prevalence of antibiotic use was higher in regional (aPR = 1.32, 95% CI: 1.23–1.42) and district hospitals (aPR = 1.31, 95% CI: 1.21–1.40) compared with the tertiary hospital. Also, the patients with an ultimately fatal McCabe score had a modest but significant increase in the likelihood of receiving antibiotics (aPR = 1.16, 95% CI: 1.04–1.30). Other demographic and clinical factors were not significantly associated with AMU (**Supplementary File 3**).

### 3.7 AMS program performance across five public hospitals in Zanzibar

AMS performance was consistently low across all hospitals, with scores ranging from 7% to 41%, and none reached the 50% functional threshold. MMH scored the highest (41%), while regional and district hospitals scored below 25%. Low scores were evident in leadership, accountability, monitoring and surveillance, and reporting and feedback, with only limited progress in the presence of DTC/IPC/AMC committees and AMS actions; many of the core elements were minimal or absent in several facilities. Reporting and feedback showed low scores, while leadership and commitment were absent in several hospitals, which indicates major gaps in AMS governance structures (**Table 5**).

**Table 5:** AMS program performance in 8 core elements indicators across 5 public hospitals in Zanzibar.

Core elements indicators	MMH	KVH	AMH	VDH	KDH
Presence of DTC, IPC, or AMS team	75.0%	50.0%	50.0%	50.0%	42.0%
DTC function	37.0%	0.0%	35.0%	15.0%	15.0%
Leadership commitment	67.0%	0.0%	0.0%	36.0%	8.0%
Accountability and responsibility	75.0%	0.0%	0.0%	25.0%	25.0%
AMS action	38.0%	19.0%	31.0%	50.0%	31.0%
Education and training	42.0%	0.0%	0.0%	58.0%	25.0%
Monitoring and surveillance	21.0%	25.0%	0.0%	25.0%	33.0%
Reporting feedback within a healthcare facility	23.0%	0.0%	5.0%	8.0%	3.0%
**Overall score**	41.0%	7.0%	14.0%	25.0%	17.0%

DTC, Drug Therapeutic Committee; ICC, Infection Control Committee; MMH, Mnazi Mmoja Hospital; KVH, Kivunge District Hospital; VDH, Vitongoji District Hospital; AMH, Abdulla Mzee Hospital; KDH, Kinyasini District Hospital. Colors are assigned automatically by the standardized WHO Healthcare Facility Core Elements Assessment Tool and indicate the level of implementation of each core element (green = high, yellow = intermediate, red = low implementation).

## 4 Discussion

The study demonstrated a very high prevalence of antibiotic use among hospitalized paediatric patients, at 87.6%, with most prescriptions being empirical in nature. This prevalence is notably higher than estimates reported from the Tanzania mainland (84.3% in children, vs. 62.3% across ages) and from other sub-Saharan African countries, including Kenya (67.7%), Botswana (70.6%), Nigeria (69.7%), and 64.0% in a systematic review (Oduyebo et al., 2017; Okoth et al., 2018; Anand Paramadhas et al., 2019; Seni et al., 2020; Boltena et al., 2024). Similarly, studies reported the prevalence of (78.2%) in Jordan (Elhajji et al., 2018), Bangladesh (91.2%) (Rashid et al., 2022), and 82.1% in Punjab and Pakistan (Sheikh et al., 2025), the level observed in Zanzibar lies at the upper extreme of global estimates. This is somewhat unexpected when compared with reports from Tanzania mainland (30.1%–62.3%) (Horumpende et al., 2020; Seni et al., 2020; Katyali et al., 2023; Zimbwe et al., 2024), which have been documented across mixed patient populations. The observed difference suggests a more intensive reliance on antibiotics in Zanzibar paediatric wards, which may be driven by limited diagnostic capacity, high burden of infectious diseases, and clinical uncertainty leading to empirical prescribing in resource-limited settings (Massele et al., 2023; Otaigbe and Elikwu, 2023). In contrast, substantially lower rates were reported in high-income settings(<45.0%) in Europe, Brazil, and Belgium (Gharbi et al., 2016; Plachouras et al., 2018; Porto et al., 2020; Vandael et al., 2020). This reflects stronger antimicrobial stewardship (AMS) systems and improved diagnostic support.

A clear hospital tier effect was observed, with near-universal prescribing at district and regional hospitals (>92%) compared to 71.8% at MMH, the tertiary hospital. However, the reverse pattern observed in this study suggests stronger empirical prescribing practices in lower-tier facilities, likely reflecting limited diagnostic capacity and fewer clinical decision-support resources. Similar patterns have been reported in secondary-level hospitals in Bangladesh (71.5%) compared with tertiary centres (60.2%) (Rashid et al., 2022)Whereas studies from high-income countries such as the UK demonstrate lower and more regulated antibiotic use across hospital tiers(from approximately 36.0% to 43.0%) (Gharbi et al., 2016). These differences highlight the need for facility-specific AMS interventions tailored to hospital level and resource availability.

Community-acquired infections (CAIs) were the most common indication for antibiotic use (60.3%), consistent with studies conducted in Tanzania (Horumpende et al., 2020; Seni et al., 2020; Kihwili et al., 2023). This finding is consistent with studies conducted in Tanzania, Uganda, and Ghana using the WHO-PPS methodology (Labi et al., 2021; Kiggundu et al., 2022) as well as multinational Global-PPS studies (Blackburn et al., 2021; D’Arcy et al., 2021). The predominance of CAIs likely reflects the high burden of infectious diseases among children in Zanzibar and suggests that empirical treatment remains the dominant management strategy. This is further reinforced by limited access to timely microbiological diagnostics, which constrains targeted therapy and promotes broad empirical antibiotic use. In contrast, higher proportions of prophylactic use reported in Europe are associated with surgical prophylaxis and more advanced procedural care systems (Plachouras et al., 2018).

Across the five hospitals surveyed, all antibiotics where prescribing was predominantly conducted medical doctors (general practitioners) (77.7%). This is in contrast to findings from Uganda and parts of mainland Tanzania, where nurses or clinical officers play a more prominent prescribing role (Okello et al., 2020; Mabilika et al., 2022). These differences reflect variability in workforce structure across health systems and hospital tiers. In contrast, high-income settings typically employ multidisciplinary AMS teams led by infectious disease specialists, pharmacists, and microbiologists, ensuring more structured prescribing practices (Reingold et al., 2023). The current findings suggest that limited specialist involvement and workforce constraints may contribute to less regulated prescribing practices in Zanzibar.

The most frequently prescribed antibiotics were gentamicin, ampicillin, and ceftriaxone. This pattern is consistent with their inclusion in national treatment guidelines, affordability, and widespread availability. Similar findings have been reported in Gambia, South Africa, and multicountry pediatric PPS studies (Hsia et al., 2019). However, differences exist compared with studies in Tanzania mainland, Uganda, and Belgium, where ceftriaxone and metronidazole are more dominant (Horumpende et al., 2020; Seni et al., 2020; Vandael et al., 2020; Kiggundu et al., 2022). Of concern, over half of all antibiotics prescribed belonged to the WHO Watch group (53.8%), indicating a relatively high reliance on antibiotics with higher resistance potential. This pattern is consistent with findings from other LMIC settings, including Pakistan, Bangladesh (Rashid et al., 2022; Mustafa et al., 2024), and sub-Saharan Africa (Hsia et al., 2019; Katyali et al., 2023). Such a high proportion of Watch antibiotics is concerning, as it may accelerate antimicrobial resistance if not carefully monitored through strengthened AMS interventions. In contrast, higher proportions of Access antibiotics reported in some settings reflect more conservative and guideline-driven prescribing practices (Seni et al., 2020; Kiggundu et al., 2022; Skosana et al., 2022; Katyali et al., 2023; Zimbwe et al., 2024).Ampicillin–cloxacillin, as recommended in the Zanzibar Standard Treatment Guidelines, was prescribed in only 7.5% of cases. This low utilization may reflect the limited availability of cloxacillin formulations and substitution with broader-spectrum alternatives. This discrepancy highlights the need for further evaluation of antibiotic availability, prescribing adherence, and potential misalignment between guidelines and clinical practice, supported by integration of AMR surveillance data.

Almost all antibiotics were administered parenterally (98.6%), which is consistent with the clinical severity of hospitalized paediatric patients. Similar findings have been reported in Tanzania and Uganda (Kiggundu et al., 2022; Katyali et al., 2023). However, the absence of reliable data on intravenous-to-oral switching limits assessment of optimal antibiotic stewardship practices, particularly de-escalation strategies, which are an important component of AMS.

The utilization of laboratory services revealed a critically low rate of culture and antimicrobial susceptibility testing (AST), with only 10.9% of children undergoing culture before antibiotic initiation. This finding indicates a strong reliance on empirical therapy in the management of paediatric infections. The low uptake of culture and AST may be partly explained by the limited availability of microbiological diagnostic services, as only two hospitals (MMH and AMH) had functional laboratory capacity during the study period. Nevertheless, similarly low levels of diagnostic utilization have also been reported in mainland Tanzania (Horumpende et al., 2020; Seni et al., 2020; Katyali et al., 2023) and Pakistan (Mustafa et al., 2024), while higher rates have been documented in settings such as Gambia (Chaw et al., 2018), highlighting considerable variation in diagnostic stewardship practices across countries. Importantly, alternative diagnostic approaches may support clinical decision-making in resource-limited settings. For example, a feasibility study conducted in Zanzibar demonstrated that serial C-reactive protein (CRP) point-of-care testing can aid in optimizing antibiotic use among hospitalized paediatric patients (Joshua et al., 2025), suggesting a potential role for simple biomarkers in strengthening antibiotic stewardship where culture and AST capacity is limited.

Antibiotic prescribing was significantly influenced by hospital tier and disease severity, particularly McCabe score. Children in regional and district hospitals were more likely to receive antibiotics compared with those in tertiary care, reinforcing the role of institutional context as a key driver of AMU. In contrast, demographic factors were not significantly associated with antibiotic use, suggesting that institutional and clinical factors play a more dominant role. This differs from findings in Tanzania mainland, where age and ward type were significant predictors (Seni et al., 2020), likely due to differences in the study population and inclusion criteria.

AMS performance was generally poor across all five hospitals, with scores below 50%, indicating weak or non-functional stewardship systems. District and regional hospitals showed particularly low performance, with minimal governance structures, surveillance systems, or feedback mechanisms. These findings are concerning, given the established importance of AMS in reducing inappropriate antibiotic use. Compared with countries such as Kenya, where AMS implementation has shown moderate progress despite resource limitations (Gitonga et al., 2025) (Joshua et al., 2025), Zanzibar faces greater challenges due to limited infrastructure, workforce shortages, and weak surveillance systems (Revolutionary Government of Zanzibar, 2019; World Health Organization, 2019). These gaps highlight an urgent need for strengthening AMS governance, capacity-building, and implementation of structured stewardship interventions aligned with the Zanzibar AMR Action Plan.

Overall, the findings provide strong evidence of high antibiotic use, substantial reliance on empirical therapy, and weak AMS implementation across hospital tiers in Zanzibar. These results underscore the need for targeted, facility-specific AMS interventions, improved diagnostic capacity, and strengthened adherence to national treatment guidelines to promote rational antibiotic use and reduce the risk of antimicrobial resistance.

### 4.1 Study strengths and limitations

The use of two standardized WHO tools (WHO-HCF AMS Assessment and WHO-PPS) enabled comprehensive evaluation of AMS capacity and AMU at the facility and patient levels, respectively. Conducting the study across multiple hospital tiers further enhanced its relevance and yielded valuable insights to strengthen hospital tier-specific AMS programs. Incomplete medical documentation in the patients’ files limited assessment of other key AMS parameters (like parenteral-oral-switch, antibiotic dosage, and co-morbidities), which in turn limited evaluation of temporal trends and comprehensive multivariate analysis.

## 5 Conclusions

This study aimed to assess antibiotic use patterns, factors influencing antibiotic use, and antimicrobial stewardship (AMS) performance among hospitalized paediatric patients in Zanzibar. The findings showed a high prevalence of antibiotic use, mainly influenced by hospital tier and disease severity, with widespread empirical prescribing, limited use of culture and antimicrobial susceptibility testing, and weak AMS implementation across all hospitals. These results highlight critical gaps in prescribing practices and AMS systems, with important implications for the potential rise of antimicrobial resistance. Strengthening AMS structures, improving diagnostic capacity, and enhancing monitoring systems are urgently needed to support rational antibiotic use in Zanzibar. The study provides evidence to guide national AMS interventions and future research on antibiotic use and resistance patterns.

## The ZanTOTO collaborators:

Timothy Walsh (The International Centre for Antimicrobial Resistance Solutions, Copenhagen, Denmark); Salim Slim (Department of Preventive Services, Ministry of Health, Zanzibar), and Abdul-Latif Haji (Directorate of Policy, Planning and Research – Ministry of Health, Zanzibar); Mwanakhamis Seif (Mnazi Mmoja Hospital, Zanzibar).

## Data Availability

The raw data supporting the conclusions of this article will be made available by the authors, without undue reservation.
