# Supplementary material for: Drivers influencing antibiotics use in pediatric wards across five public hospitals in Zanzibar: a WHO multiple point prevalence survey
Source: Front Antibiot. 2026 Jul 30;5:1874316. doi: 10.3389/frabi.2026.1874316 (PMC13470639; doi:10.3389/frabi.2026.1874316)
Supplement: Supplementary file 1 [file Table1.docx]

**Supplementary file 1: Overall distribution of antibiotics prescribed among pediatric inpatients in five public hospitals in Zanzibar**

| **Antibiotics** | **No of antibiotics** | **Percent (%)** |
| --- | --- | --- |
| Gentamicin | 494 | 35.7 |
| Ampicillin | 440 | 31.8 |
| Ceftriaxone | 223 | 16.1 |
| Ampicillin-Cloxacillin | 104 | 7.5 |
| Metronidazole | 53 | 3.8 |
| Ciprofloxacin | 20 | 1.4 |
| Amoxicillin-clavulanate | 13 | 0.9 |
| Benzylpenicillin | 17 | 1.2 |
| Others* | 21 | 1.5 |
| **Total** | 1385 | 100 |

*Others: Amoxicillin (n=3), cefixime (n=1), cefotaxime (n=1), erythromycin (n=1), meropenem (n=4), phenoxymethylpenicillin (n=3), sulfamethoxazole-trimethoprim (n=7), and vancomycin(n=1).

**Supplementary file 2: Overall distribution of diagnoses among pediatric inpatients in five public hospitals in Zanzibar**

| **Diagnosis** | **Frequency**  **(n)** | **Prevalence (%)** |
| --- | --- | --- |
| Antibiotic use for a purpose other than treatment | 297 | 33.7 |
| Pneumonia | 241 | 27.3 |
| Clinical sepsis | 159 | 18.0 |
| Gastrointestinal infections | 72 | 8.2 |
| Systemic inflammatory response without clear anatomical site | 29 | 3.3 |
| Cellulitis, wound, deep soft tissue not involving bone, unrelated to surgery | 17 | 1.9 |
| Central nervous system infections | 12 | 1.4 |
| Infection of the ear, nose, throat, larynx, and mouth | 12 | 1.4 |
| Others* | 43 | 4.9 |
| **Total** | 882 | 100 |

*Others: Septic arthritis, osteomyelitis, unrelated to surgery(n=6), Septic arthritis, osteomyelitis of surgical site(n=2), completely undefined, site with no systemic inflammation(n=2), febrile neutropenia or other infection in immunocompromised host without clear anatomical site(n=5), asymptomatic bacteriuria(n=2), cardiovascular infections, endocarditis, vascular graft(n=1), surgical site infection involving skin or soft tissue but not bone(n=4), intraabdominal sepsis, including hepatobiliary(n=2), symptomatic lower urinary tract infection(n=1).

Note. Percentages are rounded to one decimal place and may total greater than 100% due to rounding.

**Supplementary file 3: Factors influencing antibiotic use among pediatric patients admitted to pediatric wards across five public hospitals in Zanzibar**

| **Variable** | **Total** | **Patients on antibiotics**  **N (%)** | **Modified Poisson regression** | | | |
| --- | --- | --- | --- | --- | --- | --- |
|  |  |  | **Bivariate** | | **Multivariate** | |
|  |  |  | **IRR [95%CI] **** | **P-value** | **IRR [95%CI] **** | **P - value** |
| **Hospital Tier** |  |  |  |  |  |  |
| Tertiary | 333 | 239(71.77) | 1 |  | 1 |  |
| Regional referral | 163 | 162(99.39) | 1.38(1.29-1.48) | <0.001 | 1.39(1.30-1.50) | <0.001 |
| District | 447 | 425(95.0) | 1.35(1.25-1.46) | <0.001 | 1.36(1.26-1.46) | <0.001 |
| **Gender** |  |  |  |  |  |  |
| Female | 536 | 471(87.90) | 0.99(0.94-1.04) | 0.683 | 0.98(0.94-1.03) | 0.409 |
| Male | 407 | 355(87.19) | 1 |  | 1 |  |
| **Age group (months)** |  |  |  |  |  |  |
| >60 | 71 | 62(87.32) | 1 |  | 1 |  |
| 13-60 | 233 | 214(91.85) | 1.05(0.97-1.16) | 0.210 | 1.06(0.96-1.16) | 0.249 |
| 2-12 | 297 | 249(83.84) | 1.01(0.92-1.12) | 0.771 | 1.02(0.93-1.13) | 0.653 |
| ≤1 | 326 | 285(87.42) | 1.06 (0.97-1.17) | 0.212 | 1.05(0.96-1.16) | 0.278 |
| **Surgery since admission** |  |  |  |  |  |  |
| Yes | 39 | 795(87.94) | 0.98(0.84-1.14) | 0.799 | 0.99(0.85-1.15) | 0.867 |
| No | 904 | 31(79.49) | 1 |  | 1 |  |
| **Transfer from other hospitals** |  |  |  |  |  |  |
| Yes | 133 | 115(86.47 | 0.99(0.92-1.06) | 0.831 | 0.99(0.92-1.06) | 0.760 |
| No | 810 | 711(87.78 | 1 |  | 1 |  |
| **Admission in past 90 days** |  |  |  |  |  |  |
| Yes | 19 | 15 (78.95) | 1.071(0.831-1.380) | 0.593 | 1.08(0.84-6 1.37) | 0.547 |
| No | 924 | 811 (87.77) | 1 |  | 1 |  |
| **McCabe score** |  |  |  |  |  |  |
| NF non-fatal | 873 | 765(87.63) | 1 |  | 1 |  |
| RF Rapidly fatal | 3 | 2(66.67) | 0.94(0.42-2.09) | 0.873 | 0.95(0.44-2.10) | 0.903 |
| UF Ultimately fatal | 22 | 21(95.45) | 1.2(1.07-1.34) | 0.002 | 1.20(1.07-1.34) | 0.002 |

**Bivariate incidence rate ratio (IRR) adjusted for hospital type (fixed effect); **Multivariate incidence ratio (IRR), hospital type excluded in the model

IRR=PR; The study used a cross-sectional study design, so the incidence rate ratio (IRR)=Prevalence Ratio (PR)

Note: Hospital type was excluded from the final model due to collinearity, as no meaningful differences in outcomes were observed across hospitals**.**
